# Transcriptional induction of NF-κB-inducing kinase by E2F4/5 facilitates collective invasion of GBM cells

**DOI:** 10.1038/s41598-023-38996-9

**Published:** 2023-08-11

**Authors:** Kathryn M. Pflug, Dong W. Lee, Kassandra McFadden, Linda Herrera, Raquel Sitcheran

**Affiliations:** 1 Department of Cell Biology and Genetics, School of Medicine, Texas A&M University Health Science Center, Bryan, TX 77807 USA; 2 Present Address: 59Th Medical Wing, San Antonio Air Force Base, San Antonio, TX 78236 USA; 3 Present Address: Massachusetts General Hospital, 55 Fruit St., Boston, MA 2114 USA

**Keywords:** Cancer, Cell invasion, Collective cell migration

## Abstract

The prognosis of high-grade gliomas, such as glioblastoma multiforme (GBM), is extremely poor due to the highly invasive nature of these aggressive cancers. Previous work has demonstrated that TNF-weak like factor (TWEAK) induction of the noncanonical NF-κB pathway promotes the invasiveness of GBM cells in an NF-κB-inducing kinase (NIK)-dependent manner. While NIK activity is predominantly regulated at the posttranslational level, we show here that NIK (*MAP3K14*) is upregulated at the transcriptional level in invading cell populations, with the highest NIK expression observed in the most invasive cells. GBM cells with high induction of NIK gene expression demonstrate characteristics of collective invasion, facilitating invasion of neighboring cells. Furthermore, we demonstrate that the E2F transcription factors E2F4 and E2F5 directly regulate NIK transcription and are required to promote GBM cell invasion in response to TWEAK. Overall, our findings demonstrate that transcriptional induction of NIK facilitates collective cell migration and invasion, thereby promoting GBM pathogenesis.

While glioblastoma multiforme (GBM) tumors rarely metastasize outside of the central nervous system (CNS), their aggressive growth and persistent invasiveness into healthy brain tissue are major factors underlying resistance to conventional treatment methods such as surgery, irradiation, and chemotherapy^1, 2^. In addition to single-cell invasion, multicellular, connected networks of GBM cells have been observed in human tumors and mouse models^3–5^. This collective invasion, defined by the coordinated movement of cells into surrounding tissue while maintaining cell–cell junctions, is a process that occurs in epithelial regeneration and during the development and remodeling of large tissue structures, including angiogenesis and neural crest cell development^6,7^. Collective invasion has also been identified as a significant mode of GBM cell infiltration into healthy brain tissue. During collective invasion, cells acquire leader–follower phenotypes, in which both leader (pioneer) and follower cells assume different metabolic activities and work in concert to promote tumor cell dispersion. Leader cells direct the leading edge of the tumor, migrating through the microenvironment, paving the path of invasion, and transmitting information to follower cells^8, 9^. However, there is an increasing body of evidence that tumor cells in collective invasion may alternate between leader and follower phenotypes, warranting further studies of this process.

Activation of NF-κB signaling pathways has been well documented in a variety of malignancies, including GBM^10^. We previously demonstrated that activation of the noncanonical NF-κB pathway through TNF-weak-like factor (TWEAK) is a potent inducer of GBM invasion^11^. Specifically, NF-κB-inducing kinase (NIK; encoded by *MAP3K14*), an essential upstream kinase for noncanonical NF-κB activation, increases matrix metalloproteinase 1 (MT1-MMP) activity to promote invadopodia formation during GBM invasion^12^. NIK is predominantly regulated at the posttranslational level, whereby constitutive proteosome-dependent NIK protein turnover is attenuated in a signal-dependent manner, resulting in the accumulation of catalytically active NIK. In the current study, we show that the dynamic, signal-dependent upregulation of NIK at the transcriptional level is pronounced in cells at the leading edge of gliomaspheres, enhancing collective invasion of neighboring cells into the surrounding matrix.

## Results

### Induction of NIK transcription directly correlates with GBM cell invasion

**Figure 1:**
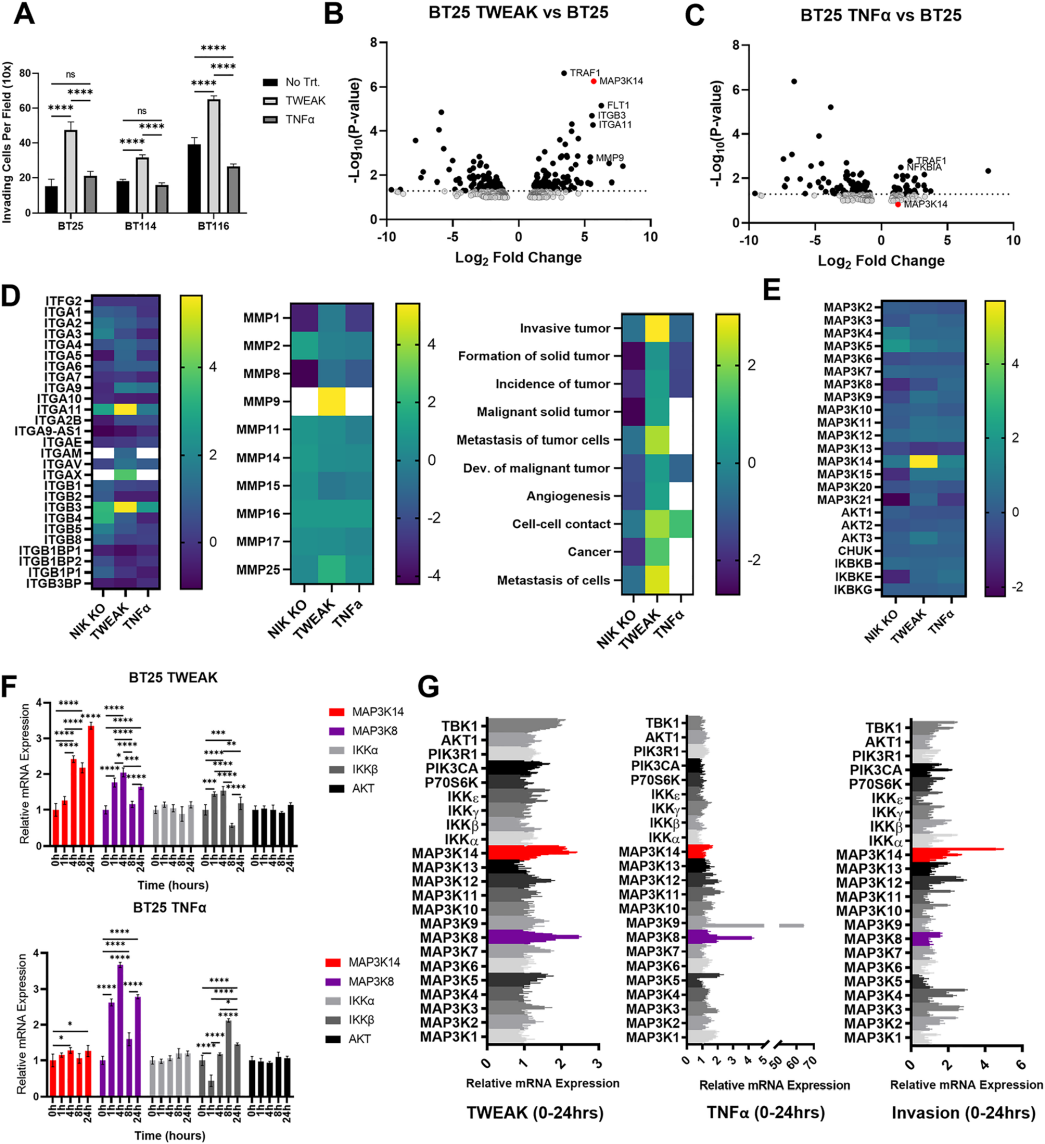
Induction of NIK transcription directly correlates with GBM cell invasion. (**A**) Quantification of three-dimensional collagen invasion assay BT25, BT114, and BT116 patient derived GBM cells after 48 h. Invasion was conducted under basal, TWEAK (10 ng/mL), or TNFα (10 ng/mL) treated conditions. Data represented as mean±SD, two-way ANOVA p < 0.001. (**B**–**E**) RNA sequencing analysis of BT25 GBM cells with either TWEAK treatment (10 ng/mL for 4 h) or TNFα treatment (10 ng/mL for 30 min) on wild-type cells or NIK KO BT25 cells compared to wild-type BT25 cell gene expression. IPA software was used for pathway analyses. (**F**) RT-qPCR analysis of BT25 cells for MAP3K14, MAP3K8, IKKα, IKKβ, AKT. Cells were treated with either 10 ng/mL TWEAK or 10 ng/mL TNFα and analyzed at 0, 1, 4, 8, and 24 h. Data represented as mean±SD, two-way ANOVA. (**G**) RT-qPCR analysis was utilized to further examine the expression of NF-κB proteins and MAP3Ks in BT114 GBM cells. Cells were treated with either 10 ng/mL TWEAK or 10 ng/mL TNFα and analyzed at 0, 0.25, 0.5, 1, 4, 8, 16, and 24 h, represented by each group of shaded bars. RT-qPCR analysis of cells extracted from the collagen matrix at 0, 0.5, 4, 8, and 24 h post invasion. Statistical analysis by two-way ANOVA of MAP3K14 and MAP3K8 across treatment groups; MAP3K14-TWEAK 0 h vs. 4 h, 8 h, or 24 h ****p ≤ 0.0001, MAP3K14-TNF 0 h vs. 24 h *p < .05 (other time points are ns), MAP3K14-Invasion 0 h vs. 0.5 h, 1 h, 4 h, 8 h, or 24 h ****p ≤ 0.0001. MAP3K8-TWEAK 0 h vs. 0.25 h **p ≤ 0.01, 0 h vs. 0.5 h, 4 h, or 8 h ****p ≤ 0.0001, MAP3K8-TNF 0 h vs. 0.25 h, 0.5 h, 1 h, or 4 h ****p ≤ 0.0001.

We previously reported that the invasive potential of GBM cells directly correlated with elevated RelB expression and NIK-dependent activation of the noncanonical NF-κB signaling pathway. Consistent with these findings^11^, we observed that the invasiveness of the human-derived glioblastoma (GBM) cell lines BT25, BT114, and BT116 was significantly induced by treatment with TWEAK, whereas treatment with TNFα did not stimulate invasion (Fig. 1A). Compared to our earlier results^11^, we noted increased expression of RelB in BT116 cells (Suppl. Fig. 1A), consistent with the increased basal invasion observed relative to BT25 cells. Transcriptome analysis of BT25 cells treated with TWEAK or TNFα, which preferentially activates the noncanonical or canonical NF-κB pathways respectively^13, 14^, revealed that the expression of NIK (*MAP3K14*) directly correlated with GBM cell invasion and was highly induced in response to TWEAK treatment but not TNFα treatment (Fig. 1B,C). Moreover, we observed TWEAK-specific upregulation of tumor necrosis factor receptor (TNFR)-associated factor 1 (TRAF1), a signaling adapter that interacts with and stabilizes NIK for activation of the noncanonical NF-κB pathway^15^. Elevated levels of integrin β3 (ITGB3), integrin subunit alpha 11 (ITGA11), matrix metalloproteinase 9 (MMP9), and Fms-related tyrosine kinase (FLT1) were also observed, consistent with increased invasive and migratory potential^16, 17^. Furthermore, only TWEAK-treated GBM cells exhibited increased expression of integrins, as well as MMP9, all of which are associated cancer markers^18–21^. Ingenuity Pathway Analysis (IPA) of groups of genes belonging to specific diseases and functions revealed that TWEAK treatment elevated the overall expression of cancer pathways, including tumor formation, invasion, and metastasis (Fig. 1D). Additionally, RNA-seq analysis demonstrated that, relative to other MAP3 kinases and upstream regulatory kinases in the NF-κB signaling pathways, TWEAK treatment had the greatest effect on induction of NIK/*MAP3K14* gene expression compared with TNF (Fig. 1E). RT-qPCR analysis verified RNA-seq results, and demonstrated TWEAK induced highest induction of *MAP3K14* expression, followed by *MAP3K8,* and to a much lesser extent *IKKB*, while TNF treatment mainly induced *MAP3K8* expression (Fig. 1F,G). Moreover, analysis of cells actively undergoing invasion in collagen matrices revealed that NIK/*MAP3K14* was the most highly upregulated gene compared to other kinases (Fig. 1G). We also observed TWEAK-induction of NIK mRNA and invasion in other GBM cell lines, as well as mouse embryonic fibroblasts (MEFs) (Suppl. Fig. 1B–D).

After observing elevated NIK transcription and invasion upon TWEAK treatment, we investigated potential paracrine effects among GBM cells during invasion. We found that BT116 cells underwent increased invasion when treated with conditioned media (Control CM) from highly invasive BT25 cells compared with unconditioned media. This increased invasion was dependent on NIK, as it was not observed when BT116 cells were cultured with conditioned media from NIK knockout BT25 cells (NIK KO CM). Furthermore, conditioned media from NIK KO cells rescued with ectopic expression of murine NIK (NIK KO-mNIK CM) restored induction of invasion (Suppl. Fig. 1E,F). Consistent with the results from media treatments, direct coculture of BT25 and BT114 cells increased the cell invasion/migration of the BT114 cells compared to cells cultured alone (Suppl. Fig. 1G). These results demonstrate that transcriptional induction of NIK is associated with elevated GBM invasiveness that is propagated by signal-specific, NIK-dependent paracrine signaling.

### NIK expression is upregulated in highly invasive GBM cells and promotes collective invasion

**Figure 2:**
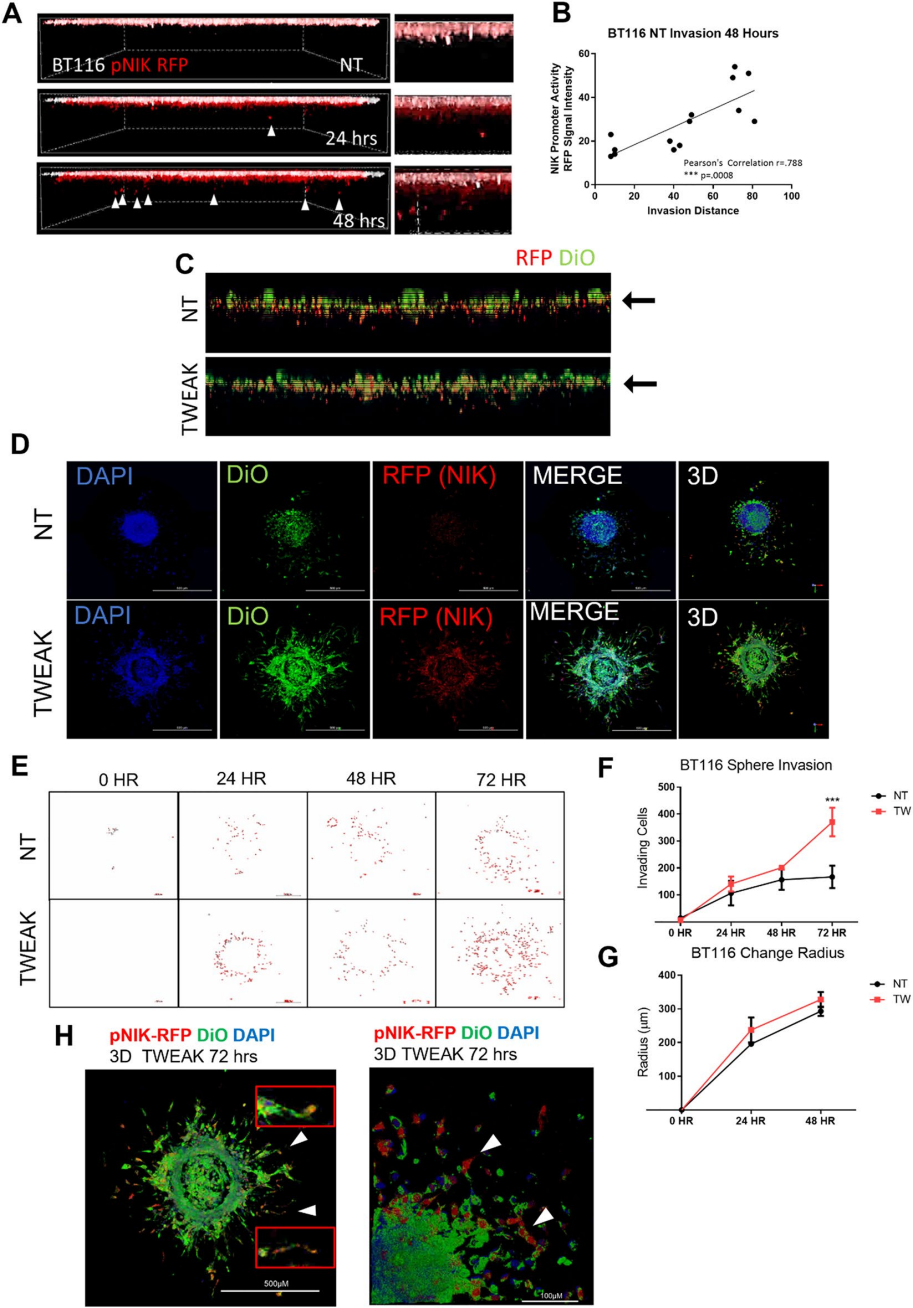
NIK expression is upregulated in highly invasive GBM cells and promotes collective invasion. (**A**) Live-cell confocal microscopy was utilized to visualize the RFP reporter under the NIK promoter (pNIK-RFP) (red) under unstimulated conditions (NT). The monolayer (0 h) was pseudocolored white and overlaid to the reference invasion distance at 24 h and 48 h. (**B**) Graph representation of RFP intensity after 48 h and distance the cells invaded. Pearson’s correlation between RFP intensity and distance is r = 0.788 and *p* = 0.008, ***p ≤ 0.001. (**C**) Invasion of BT116 pNIK-RFP cells labeled with DiO (green) with no treatment (NT) or treated with 10 ng/mL TWEAK. (**D**) BT116 pNIK-RFP cells were used for a spheroid invasion assay. Spheroids were embedded in three-dimensional collagen matrix and either left untreated (NT) or treated with TWEAK (10 ng/mL). Spheres were allowed to invade for 72 h. Confocal microscopy was used to image spheroids, labeled DiO (green), RFP reporter (red), and DAPI (blue). (**E**) Outline of BT116 pNIK-RFP (red) spheroids embedded in three-dimensional collagen for 0–72 h, either untreated (NT) or treated with TWEAK (TW) for 48 h. (**F**) The ImageJ particle analysis function was used to quantify the number of cells invading into the three-dimensional collagen matrix at various time points. Data represented as mean±SEM, two-way ANOVA. (**G**) ImageJ was used to measure the radius of the spheroids at 0 h, 24 h, and 48 h. Data represented as mean±SEM. (**H**) Three-dimensional projection image of BT116 pNIK-RFP (Red) spheroids embedded in collagen and treated with TWEAK (TW) for 72 h. Cells were labeled with DiO (green) and DAPI (blue). White arrowheads with enhanced images of cells undergoing collective invasion. Spheroid invasion assays were conducted in n > 3 either untreated or treated with 10 ng/mL TWEAK.

To monitor the induction of NIK transcription during invasion in vivo*,* we generated BT116 GBM cells stably expressing red fluorescent protein (RFP) under the promoter of NIK (pNIK-RFP). Analysis of invading BT116 pNIK-RFP cells revealed a general induction of NIK expression in invading cells (red signal from monolayer pseudocolored white) (Fig. 2A), with the farthest invading cells exhibiting the highest RFP intensity or highest expression of NIK (Fig. 2B). Treatment of BT116 pNIK-RFP cells with TWEAK further increased NIK expression (RFP signal-red/yellow), with RFP-positive cells invading farther under either untreated (NT) or TWEAK-treated conditions (Fig. 2C).

To evaluate collective invasion and leader–follower phenotypes in a 3-dimensional view, we performed invasion assays of GBMtumor spheres, which better mimic cell–cell and cell–matrix interactions. Imaging of collagen-embedded BT116 pNIK-RFP spheres revealed an increase in pNIK-RFP expression upon TWEAK treatment (Fig. 2D), with the highest pNIK-RFP expression observed among the most invasive cells at the leading edge of the sphere (Fig. 2E, Suppl. Fig. 2A). Consistent with single-cell invasion assays, the induction of NIK expression directly correlated with the migration and dispersion of cells from GBM spheres, which was enhanced upon TWEAK treatment (Fig. 2F) compared to the invasion of similar-sized spheres from the untreated group (Fig. 2G). Cells with a high pNIK-RFP signal were observed among cells undergoing collective invasion at the sphere peripheries (Fig. 2H). These results demonstrate that cells with the highest TWEAK-induced NIK expression directly correlated with the most invasive GBM cells, facilitating collective invasion, consistent with increased invasion and metastasis gene signatures.

### Inhibition of NIK activity reduces GBM cell invasion

**Figure 3:**
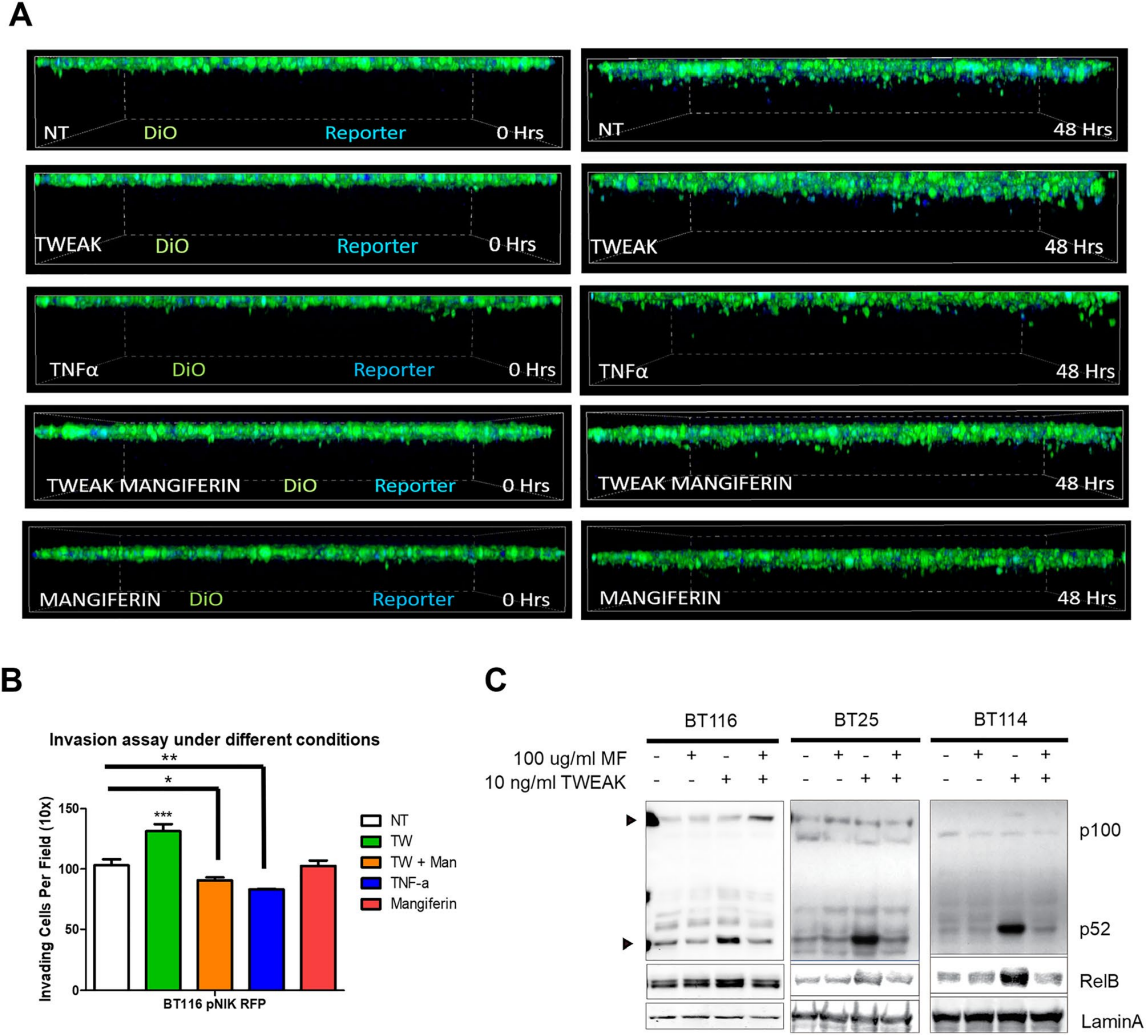
Inhibition of NIK activity reduces GBM invasion. (**A**) BT116 pNIK RFP cells were labeled with DiO before being seeded on collagen matrix and allowed to invade for 48 h. Live cell confocal microscopy was used to obtain Z-stack images over 48 h. Cells either received no treatment (NT) or were treated with 10 ng/mL TWEAK, 10 ng/mL TNF-α, 10 ng/ml TWEAK with 100 μg/mL mangiferin, or 100 μg/mL mangiferin. (**B**) Quantification of cell invasion after 48 h. Data represented as mean±SEM, one-way ANOVA. (**C**) Immunoblot showing decreased RelB and p52 in the nucleic fraction of GBM cells treated with mangiferin.

Next, we evaluated whether NIK catalytic activity was required to promote GBM invasion. Treatment of cells with mangiferin, a natural inhibitor of NIK^22–24^, significantly attenuated TWEAK-stimulated invasion to levels comparable with untreated or TNF-treated conditions (Fig. 3A,B). Mangiferin was verified to inhibit activation of the noncanonical NF-κB pathway by TWEAK treatment in GBM cells, as seen with a reduction in p100-p52 processing and RelB (Fig. 3C). Mangiferin also inhibited TWEAK-induced NIK transcription (Suppl. Fig. 2B). Although mangiferin inhibited NIK and the noncanonical NF-κB pathway, it did not affect cell proliferation (Suppl. Fig. 2C). These data suggest therapeutic potential of inhibiting NIK to attenuate GBM invasion.

### E2F4 and E2F5 regulate NIK gene expression

**Figure 4:**
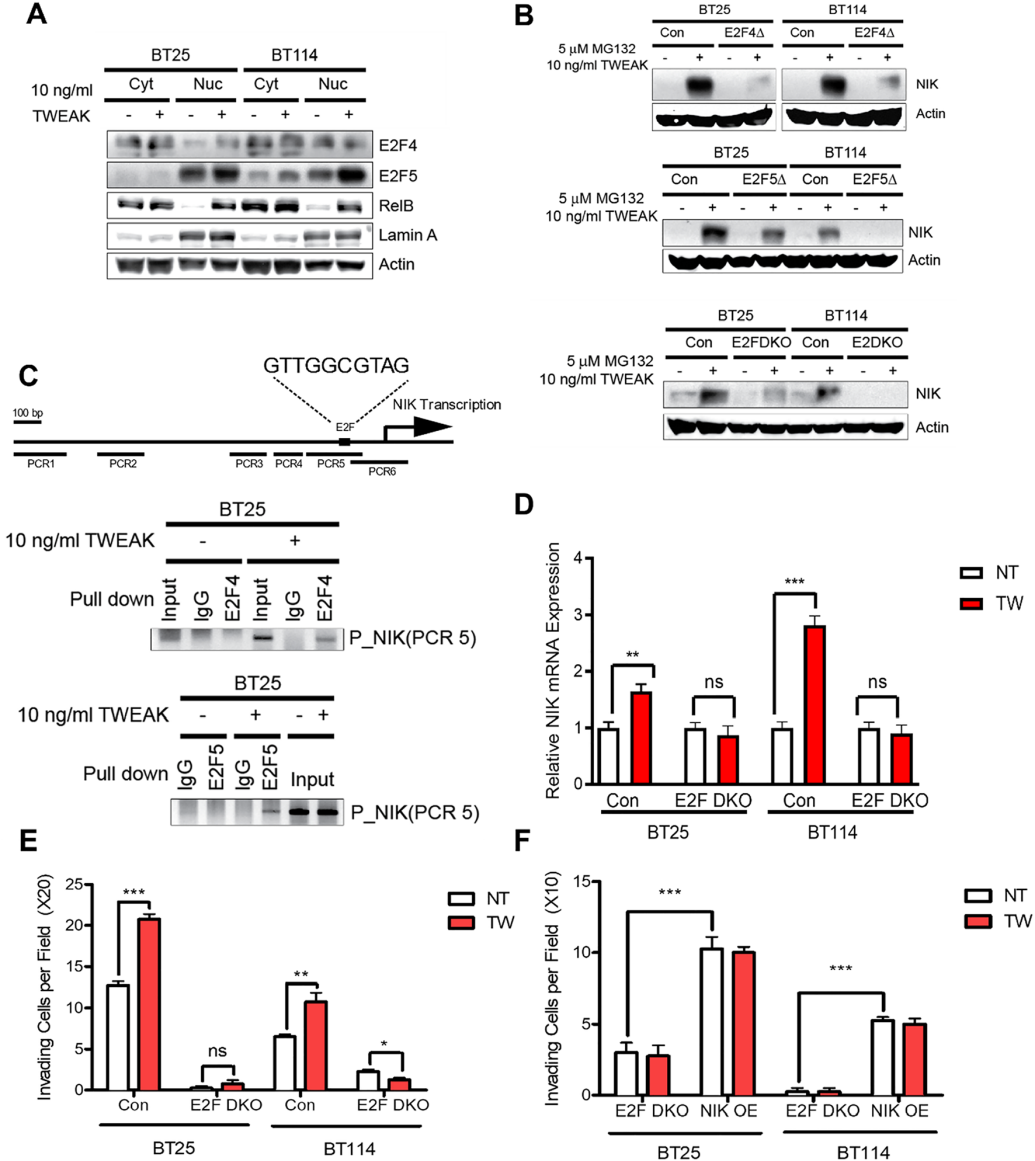
E2F4 and E2F5 regulate NIK gene expression. (**A**) Cytoplasmic and nuclear E2F4 and E2F5 were examined in GBM cells by Western blot in unstimulated vs stimulated conditions (10 ng/mL TWEAK for 4 h) with RelB as a positive TWEAK-inducible control and Lamin A as a nuclear marker. (**B**) Immunoblot of NIK expression under MG132 (5 μM)- and TWEAK (10 ng/mL)-stimulated conditions in control and E2F4, E2F5, or E2F4 and E2F5 knockout cells. **C)** ChIP analysis performed in GBM cells treated with or without 10 ng/mL TWEAK by using E2F4, E2F5, or IgG antibodies. (**D**) qPCR analysis of NIK (*MAP3K14*) induction in double knockout E2F4 and E2F5 cells (BT25 and BT114). Data represented as mean±SD, unpaired student t-test. (**E**) Invasion assay of BT25 and BT114 double knockout E2F4/E2F5 cells. Data represented as mean±SEM, unpaired student t-test. (**F**) Invasion assay of BT25 and BT114 double knockout E2F4 and E2F5 cells rescued by NIK overexpression. (**D**–**F**) Comparisons were made using glioma cells in untreated (NT) vs. TWEAK-treated (10 ng/mL for 4 h) conditions. Data represented as mean±SEM, unpaired student t-test.

The mechanisms underlying the regulation of NIK gene expression are not fully understood. A recent study identified *MAP3K14* (NIK) as a significantly upregulated gene in human osteosarcoma cells with E2F activation^25^, and analysis of the NIK promoter revealed the presence of E2F binding sites (Suppl. Fig. 3D). Thus, we next investigated whether E2F transcription factors played a role in early NIK gene expression in response to TWEAK treatment in GBM cells. We observed that TWEAK treatment increased nuclear E2F4 and E2F5 protein levels, with E2F4 having greater nuclear translocation in BT25 cells and E2F5 in BT114 cells (Fig. 4A). We also found that overexpression of E2F5, but not E2F1, increased NIK transcript levels (Suppl. Fig. 3A). E2F4 and E2F5 single and double knockout cell lines generated by CRISPR-Cas9 gene editing exhibited a significant reduction in NIK protein levels when treated with TWEAK and MG132 to inhibit proteosome-dependent degradation (Fig. 4B, Suppl. Fig. 3B). Moreover, E2F4/5 double knockout cells (E2F DKO) exhibited reduced p52/RelB nuclear translocation, demonstrating impaired activation of NIK-driven noncanonical NF-κB signaling (Suppl. Fig. 3C).

Next, we investigated whether E2F proteins directly regulate NIK transcription. Chromatin immunoprecipitation (ChIP) analyses demonstrated that antibodies specific to E2F4 and E2F5, but not IgG, were bound to certain regions of the NIK promoter (Fig. 4C, Suppl. Fig. 3D,E). Additionally, qPCR analysis of E2F-DKO cells showed significantly attenuated induction of NIK mRNA expression even with TWEAK treatment (Fig. 4D). Reduced NIK expression in E2F DKO cells proved to have functional consequences, as these cells were poorly invasive, even after TWEAK treatment (Fig. 4E), which was restored with ectopic expression of NIK (NIK OE) in E2F DKO cells (Fig. 4F). Overall, these data establish a novel role for E2F regulation of NIK transcription in a stimulated state and thus affect the cell’s overall ability to invade (Suppl. Fig. 3F).

## Discussion

While posttranscriptional regulation of NIK protein stability is important for controlling the activation of NF-κB signaling, in this study, we report that signal-specific transcriptional upregulation of NIK is strongly associated with enhanced collective invasion of GBM cells (Fig. 1). RNA-seq and qPCR analyses demonstrated that NIK induction occurred early (≤ 4 h) and was sustained with TWEAK treatment as well as in invading cells but occurred to a significantly lesser extent with TNFα at later time points (24 h). Using a NIK promoter-controlled reporter construct (pNIK-RFP) as a readout for NIK gene expression levels, we demonstrated that TWEAK-induced NIK transcription directly correlates with cell invasion and the acquisition of leader–follower cells seen in collective invasion (Fig. 2H). Indeed, we observed that the most invasive cells expressed the highest levels pNIK-RFP, suggesting a direct correlation between increased NIK gene expression and invasive potential (Fig. 2). Our data showing that conditioned media from NIK KO GBM cells was unable to stimulate invasion, while conditioned media from NIK KO-mNIK cells rescued invasion, suggests that NIK-dependent paracrine signaling propagates a collective leader–follower cell phenotype during cell invasion. Furthermore, although prior studies have shown that E2F regulates cIAPs, and E2F1 directly promotes *MAP3K14* gene expression in osteosarcoma cells^25–27^, we report for the first time that the E2F transcription factors, E2F4 and E2F5, play a key role in regulating NIK transcription and expression in GBM cells. Overall, these data reveal critical roles for the regulation of NIK at the transcriptional level in propagating collective cell invasion.

 We demonstrate that NIK promotes invasion in a noncanonical NF-κB-dependent manner in response to treatment with TWEAK. However, this does not exclude possible involvement of the canonical NF-κB pathway in regulating cancer invasion through independent or complementary mechanisms. Furthermore, we have previously demonstrated that NIK promotes mitochondrial fission and trafficking to the periphery of GBM cells during cell migration in a manner that is independent of IKKα/β and downstream NF-κB signaling^28^, suggesting that increased NIK transcription in lead invading cells may facilitate mitochondrial energy dynamics that support collective invasion.

A hallmark of invasion is alteration of the extracellular matrix to facilitate cell mobility, which includes changes in the dynamics of such proteins as e-cadherins, integrins or matrix metalloproteinases. In conjugation to promoting invasion, NIK has also been shown to regulate matrix metalloproteinase 14 (MMP14) by reducing phosphorylated MMP14, or its active form, seen in cells lacking NIK, where the opposite held true in cells expressing a constitutively active form of NIK^12^. RNA-seq analysis revealed elevated MMP9 expression in glioma cells stimulated with TWEAK, which has been associated with cancer biomarkers^18^. Furthermore, as collective invasion maintains cell-to-cell contact through the extracellular matrix, it has been shown that stabilization of integrins is associated with increased collective invasion of solid tumors^29^, coinciding with an increase in integrin alpha 11 and integrin beta 3 gene expression in highly invading, TWEAK-treated cells. Integrin alpha 11 and integrin beta 3 have also been linked to increased tumor progression in other cancer types^19–21^. RNA-seq disease pathway analysis also highlighted TWEAK-treated glioma cells as having higher activation of cell–cell junctions and tumor development, invasion, and metastasis. Elevated matrix metalloprotease, integrin, and tumor progression is consistent with a leader phenotype displayed by NIK-expressing cells during collective invasion.

NIK and NF-κB dysregulation are highly correlated with the induction of disease and malignancies^30–32^. Several studies have demonstrated an increase in NIK expression in various cancer models. Of these studies, increased NIK expression was observed in breast cancer, lymphomas, pancreatic cancer, gastric cancer, and GBMs^11, 33–36^. Our lab has demonstrated that NIK has a role in promoting brain tumor cell invasiveness and tumor growth in vivo through regulation of mitochondrial dynamics and metabolic homeostasis^28, 37^. Given the pro-tumorigenic role NIK has in cancer and our studies suggesting that upregulation of NIK gene expression by cytokines in the tumor microenvironment can robustly trigger invasion, the inhibition of NIK may prove a promising therapeutic target for primary as well as recurrent, invasive or metastatic tumors^22, 38–40^.

## Materials and methods

### Reagents

TNFα (ProSpec CYT-223) and TWEAK (PeproTech 31006) were used at 10 ng/mL, mangiferin was obtained from Mangiferia indica leaves (M3547-100MG, Sigma Aldrich), collagen type I (354249) was purchased from Corning, and DiD and DiO were purchased from Invitrogen.

### Cell lines

BT25, BT114 and BT116 cell lines were obtained from human GBM patients as previously described^41^. These cell lines were maintained as spheroids in neural stem cell medium containing DMEM/F-12, 1 × B-27 supplement minus vitamin A, 1 × GlutaMAX, 25 ng/mL EGF, 25 ng/mL basic fibroblast growth factor (bFGF), and 1 × penicillin/streptomycin (Life Technologies).

### Plasmids

For NIK overexpression, mouse (mNIK) cDNA was cloned into pLenti6-V5-DEST (Addgene, Cambridge, MA) using the GATE-WAYTM Cloning System (Invitrogen). For pNIK-tagRFP, the promoter of NIK was purchased from Genecopoeia (HPRM17530) and cloned into a lentiviral plasmid.

### CRISPR-Cas9 gene knockout

BT25, BT114, and BT116 cells were transduced with a mixture of LentiCrispR-v2 carrying three gRNAs for each target. The gRNA sequences for human NIK E2F4 and E2F5 are shown in Supplemental Table 1.

### RNA sequencing

1 × 10^6^ BT25 GBM cells harvested in untreated, TWEAK-treated (10 ng/mL for 4 h) and TNFα-treated (10 ng/mL for 30 min) conditions along with stable transgenic BT25 NIK KO cells. Duplicates of cell pellets were shipped on dry ice to Azenta by Genewiz (South Plainfield, NJ, USA), where RNA was isolated, and they conducted sequencing and constructed the sequencing library. For sequencing, HiSeq 2 × 150 bp was used. Sequence reads were trimmed using Trimmomatic v.0.36. The trimmed reads were mapped to the Homo sapiens GRCh38 reference genome using the STAR aligner v.2.5.2b. Unique gene hit counts were calculated by using featureCounts from the Subread package v.1.5.2. The hit counts were summarized and reported using the gene_id feature in the annotation file. Only unique reads that fell within exon regions were counted. DESeq2 files were uploaded to Ingenuity Pathway Analysis software (IPA; QIAGEN, Germantown, MD, USA) for dataset comparisons and to analyze altered genes that were statistically significant compared to wild type. IPA software was also used to analyze overall changes in disease function pathways related to identified gene families. GraphPad Prism (San Diego, CA, USA) was used to generate volcano plots and heatmaps.

### Immunoblot assays

Cells were lysed in RIPA lysis buffer (Pierce, #89900, Rockford, IL) with a protease/phosphatase inhibitor cocktail (Thermo Scientific). Equal amounts of protein were mixed with NuPage 4X LDS sample buffer (Invitrogen, NP0008) containing reducing agent and denatured at 100 °C for 7 min. Proteins were separated by 8–12% SDS-PAGE and transferred to nitrocellulose membranes (Bio-Rad, #162- 0115). The membranes were blocked for 1 h with 5% nonfat dry milk in 0.1% Tween-20/TBS (TBST) or Odyssey blocking buffer (LI-COR Biosciences, 927-40000) and incubated with primary antibodies from Cell Signaling Technology (CST) or Santa Cruz (SC): NIK CST4994, RelB CST4922, Lamin A CST 4777, NFKB2 CST4882, E2F3 SC56665, E2F4 SC69686 E2F5 SC374268, and Actin SC69879.

### Immunofluorescence staining

Collagen-embedded spheroids were seeded on eight-well chamber slides (#80827, Ibidi, Munich, Germany) or 96 half-area well plates and allowed to adhere for 2 h. During spheroid and monolayer invasion live cell imaging, cells were labeled with DiO or DiD for 30 min at 37 °C before washing three times in media. Cells were allowed to invade for 48 to 72 h, fixed with 4% paraformaldehyde, and permeabilized for 20 min with 0.3% Triton X-100 in PBS. Cells were incubated overnight in 0.1% Triton X-100 and 1% BSA in PBS at 4 °C. Cells were then incubated in 1% BSA for 1 h at room temperature. Cells were counterstained with the nuclear stain DAPI (Invitrogen, P36931).

### Three-dimensional collagen invasion assay

Monolayer invasion assays were performed as previously described^28^. Briefly, collagen type I (Corning, NY) was diluted to 2 mg/ml in DMEM/F-12 medium (1 × Pen/Strep), and matrices were polymerized in 96-well plates. A total of 4 × 105 cells cultured in NSCs or NSCs + 10% serum were seeded in triplicate in 100 μl DMEM/F-12 (1 × Pen/Strep, 1 × Glutamax) without growth factors or serum. Cells were fixed with 3% glutaraldehyde solution after 48 h of invasion and stained with 0.1% toluidine blue. Invasion density was quantified by counting cells below the plane of the monolayer by bright-field light microscopy using a 10 × 10 ocular grid at 10 × or 20 × magnification corresponding to a 1 mm 2 field. Numbers in at least three equivalent, random fields were counted (n = 3 wells each) and normalized to the corresponding control. All experiments were performed at least three times.

Live cell invasion assays were performed using GBM cells. The cells were collected and centrifuged at 1.0 rcf for 2.5 min, and the medium was removed and disassociated with accutase for 9 min at room temperature before centrifugation at 1.0 rcf for 2.5 min. Accutase was removed, and cells were resuspended in NSC media and then quantified. Approximately 2.0 × 10^6^ cells were transferred into a 15 mL conical tube, and media was added to 2 mL with 12 µL of DiO added. Cells were incubated for 30 min at 37 °C with DiO, followed by centrifugation at 1.0 rcf for 2 min. Cells were washed three times in NSC media before requantification. Cells were either used for monolayer invasion assay at 40,000 cells/well or 1.2 × 10^6^ cells were incubated for one week at 37 °C in NSC media before being embedded into 2.0 mg/ml collagen matrix.

After spheroid formation, spheres were collected at 1.0 rcf for 2.0 min and resuspended in 2 mL of fresh media. The collagen matrix was prepared, approximately 60 µL of resuspended spheres was added to the matrix, and 18 µL of spheres embedded in collagen was added to each well. Collagen was allowed to solidify for 2 h at 37 °C before taking initial images. Images were acquired over a 72-h time course.

### Image acquisition

Images were acquired with a Nikon TI A1R inverted confocal microscope with a CFI60 Plan Apochromat Lambda 10 × objective lens. Images were acquired with the following scan parameters: a "frame" scan mode of 1024 × 1024 pixels with a 16 bit depth and a grating of 3 rotations. Three-dimensional projections were obtained through Z stack images with 0.4700 µm between each image.

### RNA isolation, cDNA synthesis, and quantitative RT-PCR

Total RNA was isolated from cells by a Purelink™ RNA Mini Kit (Life Technologies). cDNA was synthesized from 1 μg of total RNA using iScript reverse transcription supermix (Bio-Rad, Hercules, CA) following the manufacturer's protocol. Quantitative RT-PCR was performed using iTaq Universal SYBR Green Supermix (Bio-Rad) with a StepOnePlus Real-Time PCR System (Applied Biosystems, Foster City, CA). The primers used are listed in Supplemental Table 2. The expression of mRNA was normalized to GAPDH expression levels. All experiments were performed at least three times with three replicates per sample.

### Supplementary Information

Supplementary Information.

## Data Availability

All data generated or analyzed during this study are included in this published article (and its Supplementary Information files).
