# Supplementary material for: Transcriptional induction of NF-κB-inducing kinase by E2F4/5 facilitates collective invasion of GBM cells
Source: Sci Rep. 2023 Aug 11;13:13093. doi: 10.1038/s41598-023-38996-9 (PMC10421885; doi:10.1038/s41598-023-38996-9)

Supplementary Figures for Pflug KM, et al. Transcriptional Induction of NF-κB-Inducing Kinase by E2F4/5 Facilitates Collective Invasion of Glioma Cells

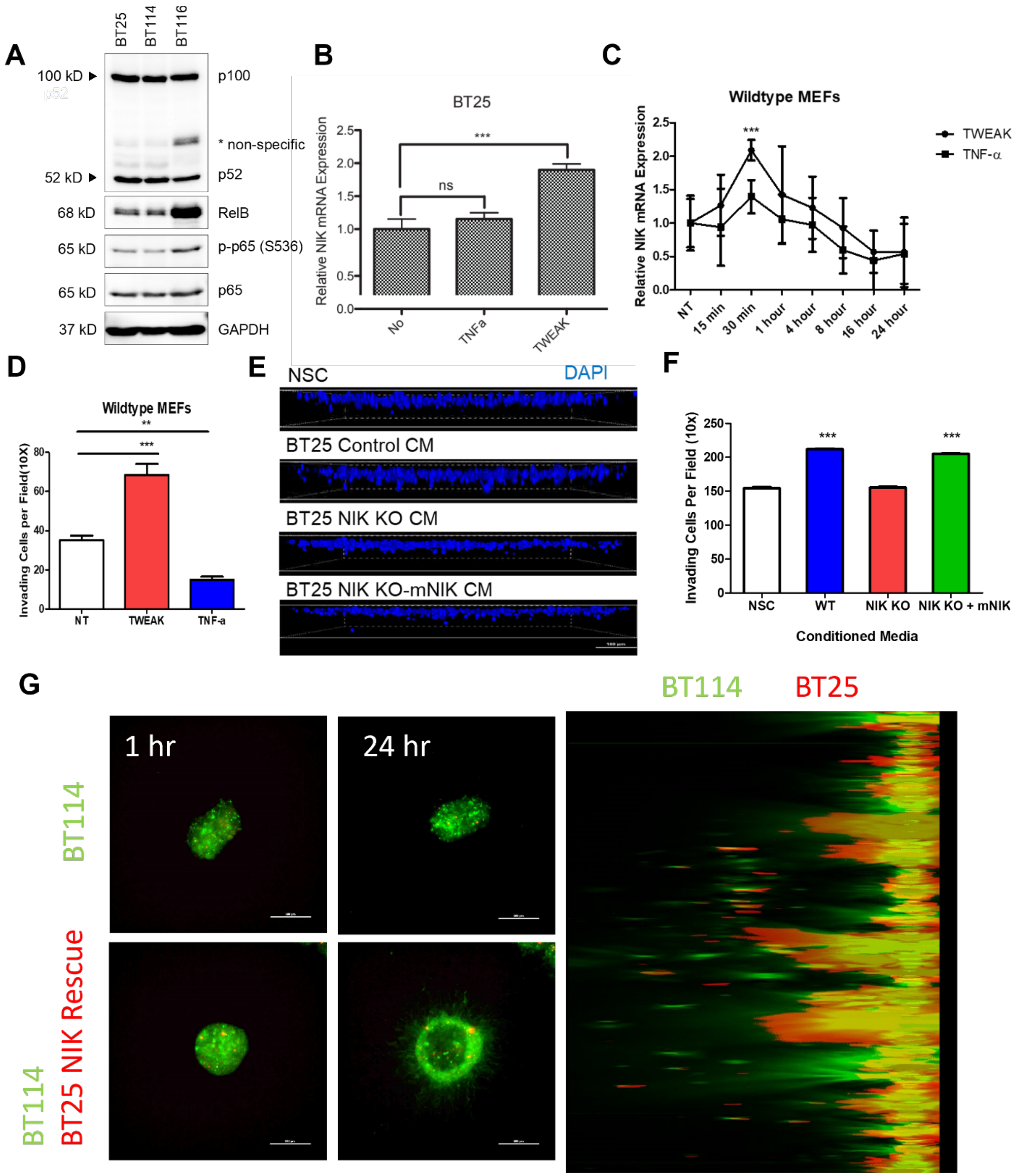

**Supplementary Figure 1: A)** Immunoblot analysis of NF-κB proteins in glioma lines BT25, BT114, and BT116. RT-qPCR analysis of relative NIK mRNA expression in **B)** BT25 cells untreated (NO) or treated with TWEAK (10ng/mL for 4 hours) or TNFα (10ng/mL for 30 minutes) and **C)** MEFs treated with TWEAK or TNFα under similar conditions. **D)** Collagen invasion assay of MEFs treated with either TWEAK or TNFα. **E)** BT116 glioma cells were stimulated with 25% conditioned media obtained from BT25 control, BT25 NIK KO, and BT25 NIK KO +mNIK (Rescue with murine NIK) cells added to normal growth media (NSC). Cells were counter stained using Hoechst for visualization. **F)** Quantification of cells that were allowed to invade into a three-dimensional collagen matrix for 48 hours before quantification and analysis. One-way ANOVA statistical analysis was used to determine significance, $p \leq 0.0001$. **G)** 3D collagen embedded glioma spheres at 1 and 24hrs of either BT114 cells alone or with BT114/BT25 NIK overexpressed (OE) cocultured.

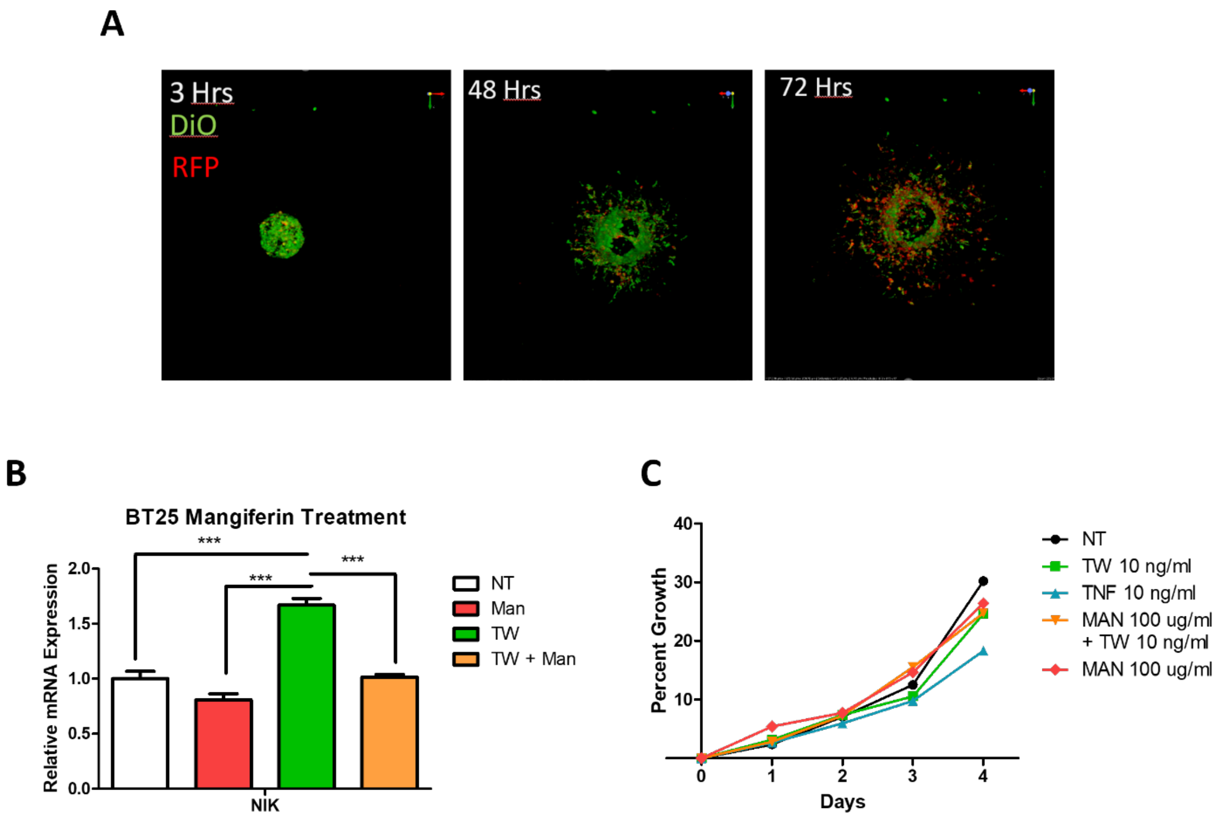


**Supplementary Figure 2: A)** Microscopy of collagen embedded glioma, BT116-pNIK-RFP spheres after 3, 48, and 72 hours of TWEAK (10ng/mL) treatment. All cells were pre labeled with DiO (green) and NIK RNA induction is shown by RFP in red. **B)** qPCR analysis of relative NIK mRNA in BT25 glioma cells treated with TWEAK, Mangiferin, or both. **C)** Cell proliferation assay of BT116 pNIK-RFP cells untreated or treated with TWEAK, TNFα, and/or Mangiferin.

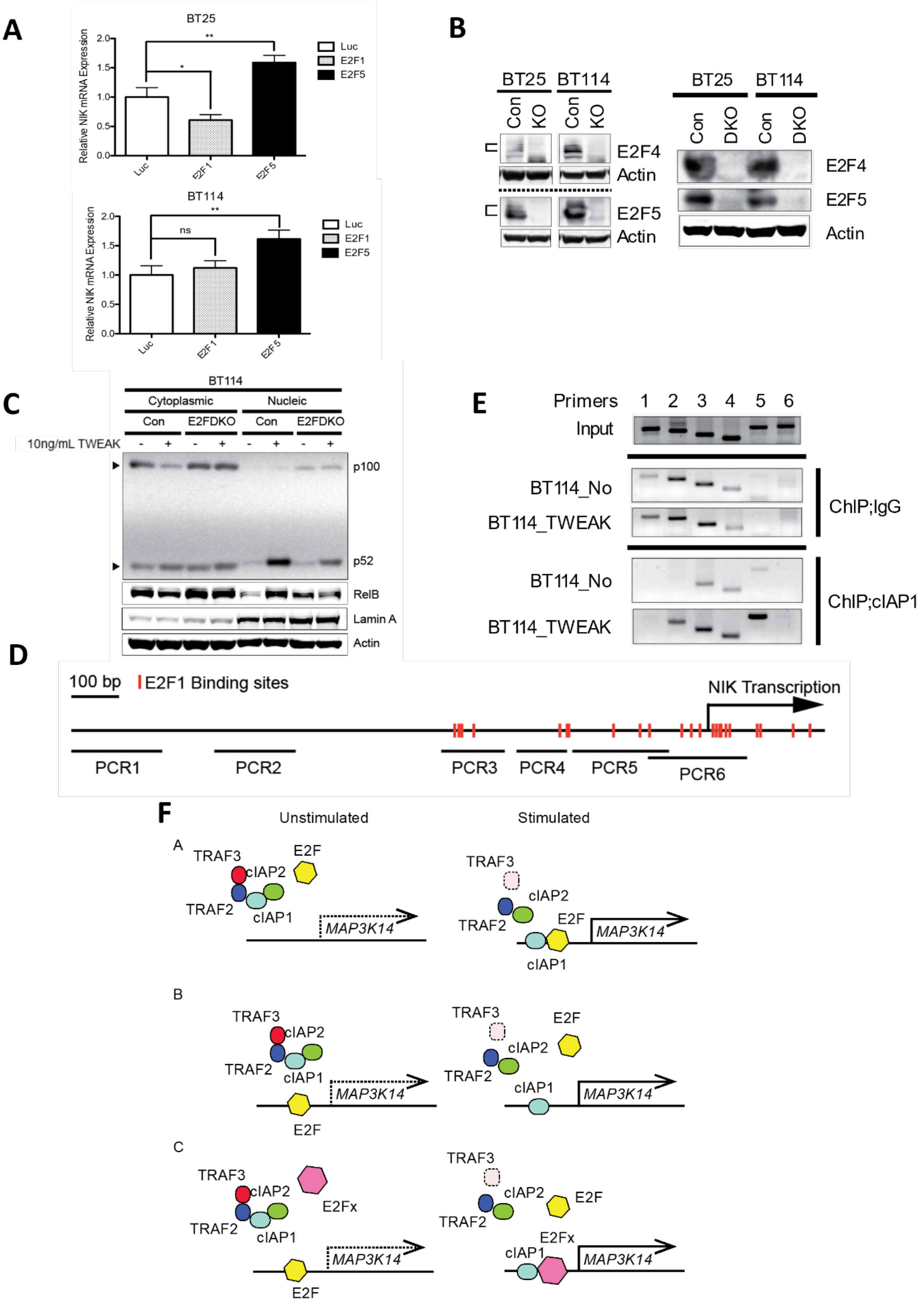

A
BT25
Relative NIK mRNA Expression
2.0
1.5
1.0
0.5
0.0
Luc
E2F1
E2F5
**
*
BT114
ns
**
B
BT25
BT114
Con
KO
DKO
E2F4
E2F5
Actin
C
BT114
Cytoplasmic
Nucleic
E2FDKO
10ng/mL TWEAK
p100
p52
RelB
Lamin A
Actin
D
100 bp
E2F1 Binding sites
NIK Transcription
PCR1
PCR2
PCR3
PCR4
PCR5
PCR6
E
Primers
1
2
3
4
5
6
Input
BT114_No
BT114_TWEAK
ChIP;IgG
ChIP;cIAP1
F
Unstimulated
Stimulated
TRAF3
TRAF2
cIAP2
cIAP1
E2F
E2Fx
MAP3K14

**Supplementary Figure 3: A)** qPCR analysis of relative NIK mRNA in BT25 and BT114 glioma cells between luciferase (Luc) transduced control or cells stably overexpressing E2F1 or E2F5. **B)** Nuclear translocation of RelB and NFKB2(p100/p52) was examined from BT25 and BT114 glioma cells. Control and E2F4/5 double knockout construct were treated with or without 10 ng/mL TWEAK for 4 hours. **C)** Model illustrating binding sites of E2F using PROMO database to identify binding sites of putative transcription factors. **D)** Models demonstrating potential E2F interaction with NF-κB proteins in unstimulated and stimulated states for NIK gene (*MAP3K14*) transduction.

| GENE | GUIDE RNAs |
|---|---|
| NIK | gNIK-1: 5′-GCUCCUUCGG AGAGGUGCAC-3′<br>gNIK-2: 5′-GAAAGCGUCGCAGCAAAGCC-3′<br>gNIK-3: 5′- AGUACCGAGAAGAAGUCCAC-3′ |
| E2F4 | gE2F4-1: 5′-UGUCGUAAAUCCGCCGCUUC-3′<br>gE2F4-2: 5′-GAAAUCUUUGAUCCCACACG-3′<br>gE2F4-3: 5′-GCACGUCCUCUGUGACGUUC-3′ |
| E2F5 | gE2F5-1: 5′-ACCUCCAGUUGUGUACCAGA-3′<br>gE2F5-2: 5′-UGAACAGGCUGCUGAUACUU-3′<br>gE2F5-3: 5′-GGCACCUUCUGGUACACAAC-3′ |

**Table 1:** Guide RNA sequences for CRISPR- Cas9 knockout of specific genes.

| GENE | PRIMERS |
|---|---|
| Human NIK (MAP3K14) | 5’-CTAGTGCATGCTCTGCAAGG-3’ and 5’-TGAGTTTCTCAGTGAGCAGGA-3’ |
| Mouse NIK (map3k14) | 5’-CCAGAATCGTCCCTCTCTAT-3’ and 5’-GACAGCCCATTTGCTTTATG-3 |
| Human MAP3K1 | 5’-GGAGACAGCCCAGACAATAAA’-3’ and 5’- ACCCGGAGCATCACAAATAG-3’ |
| Human MAP3K2 | 5’-CTGAGACCAGCAAGGAAGTAAA-3’ and 5’-CTGAGACCAGCAAGGAAGTAAA-3’ |
| Human MAP3K3 | 5’-TCGTGCAGCCATGGTTATT-3’ and 5’-TCGTGCAGCCATGGTTATT-3’ |
| Human MAP3K4 | 5’-ATCAGCAGTGCCCATGATAC-3’ and 5’- TGACGACTCAGGGACCTAAA-3’ |
| Human MAP3K5 | 5’-CCCAGAGAGAGACAGCAGATA-3’, and 5’-CTCACTGAAAGAGCCCAGATAC-3’ |
| Human MAP3K6 | 5’-CCAAAGAGCTCCGGCTAATA-3’ and 5’-CCAAAGAGCTCCGGCTAATA-3’ |
| Human MAP3K7 | 5’-CGCCTGGTACAGGAACATAAA-3’ and 5’-TGCTGCTGACTTCTGATGAC-3’ |
| Human MAP3K8 | 5’-GAGGTACCATGGTTGTCATCAG-3’ and 5’-CCTGTGGTCGTTGTCCATAAA-3’ |
| Human MAP3K9 | 5’-CCGCCCATTCAGTTGTTAGA-3’ and 5’-CCTATCCAGAAAGCACGATAGAC-3’ |
| Human MAP3K10 | 5’- GGCACAAGACCACCAAGAT-3’ and 5’- GAGAAGAGGGAGAGACGGATAA-3’ |
| Human MAP3K11 | 5’- CCCTGAAGATCACCGACTTT-3’ and 5’- AAGGTGGAGGCCTTGATAAC-3’ |
| Human MAP3K12 | 5’- GCGCCACATAATCAACAGAAAG-3’ and 5’- GCCAGTGTCCCTAGAGTTTATC-3’ |
| Human MAP3K13 | 5’- CAGCTAGAAATGCGGGAGAA-3’ and 5’- TGGCATTGGGATGGATGATAG-3’ |
| Human PI3K | 5’- GGGCTTTCTGTCTCCTCTAAAC-3’ and 5’- ATGTCTGGGTTCTCCCAATTC-3’ |
| Human AKT | 5’- CTACAACCAGGACCATGAGAAG-3’ and 5’- TCTTGAGCAGCCCTGAAAG-3’ |
| Human S6K | 5’- CAAGGTGAGGGAGATAGGGATA-3’ and 5’-AAGGAAGGTAGACAGCAGAAAC-3’ |
| Human IKKα | 5’- GTCAGGGAGACTTGATGGAATC-3’ and 5’- CATCTCTGTGCTGTCACTGTAG-3’ |
| Human IKKβ | 5’- TCTCCTGCTGATTGTGTGTG-3’ and 5’- CTTGCCTTTCGGGTGTTATTTC-3’ |
| Human IKKγ | 5’-TCACCAGCTCTTCCAAGAATAC-3’ and 5’- CTGGAGCTGCTGTTTGAGAT-3’ |
| Human IKKε | 5’-TCCCACTCCCTCTGGTTTAT-3’ and 5’- AGACTGTGATGAGGTCGTTTG-3’ |
| Human TBK1 | 5’-GAAGGGCCTCGTAGGAATAAAG-3’ and 5’-CCCGAGAAAGACTGCAAGAA-3’ |
| Human PIK3R1 | 5’- GCTTTGCCGAGCCCTATAA-3’ and 5’-ACATTGAGGGAGTCGTTGTG-3’ |
| Human GAPDH | 5’-GAAGGTGAAGGTCGGAGTC-3’ and 5’-GAAGATGGTGATGGGATTTC-3’ |

**Table 2:** Primers for various kinases for qPCR analysis in glioma cells.

UNCROPPED GELS.

## Figure 3C

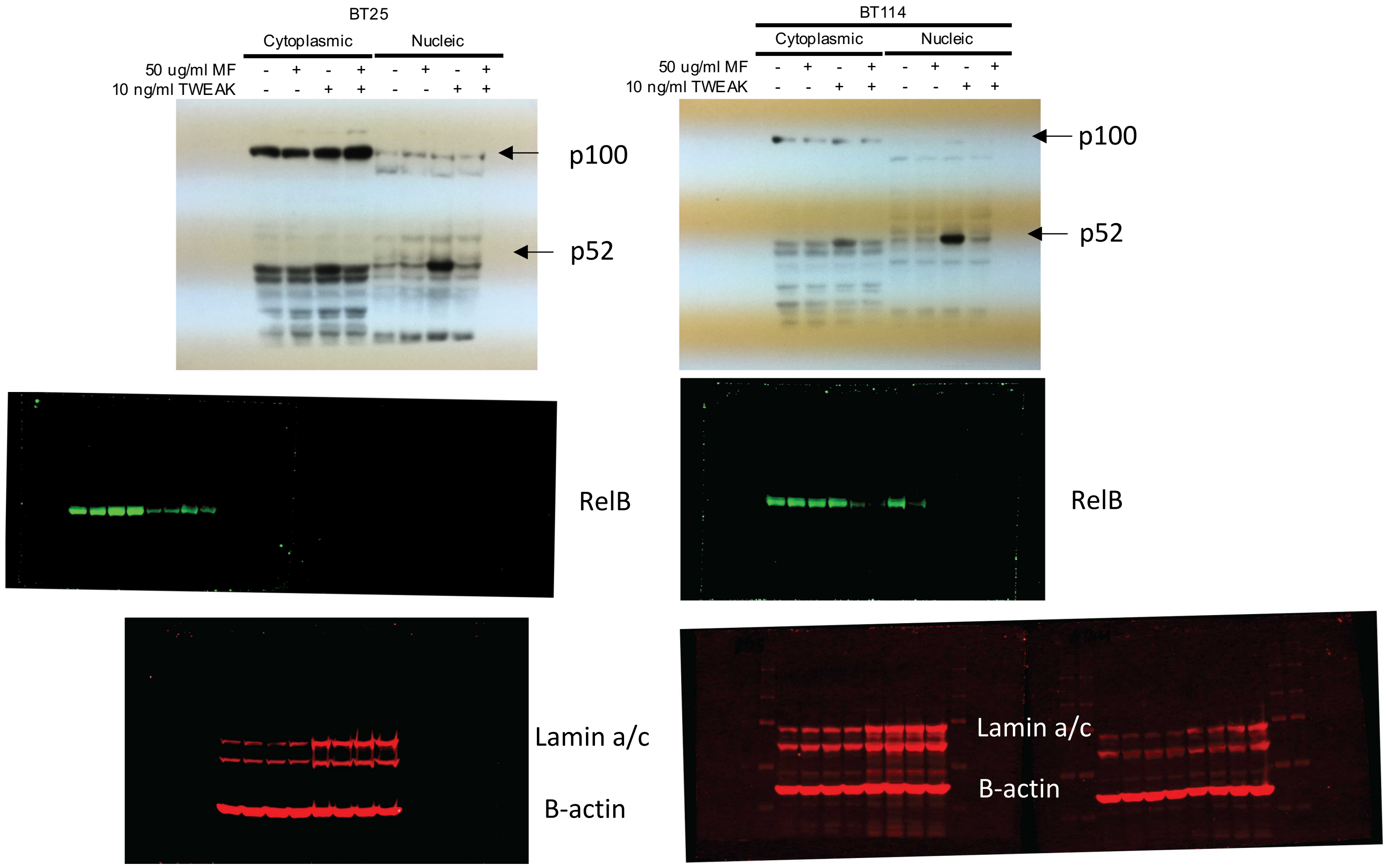

Figure 3C continued

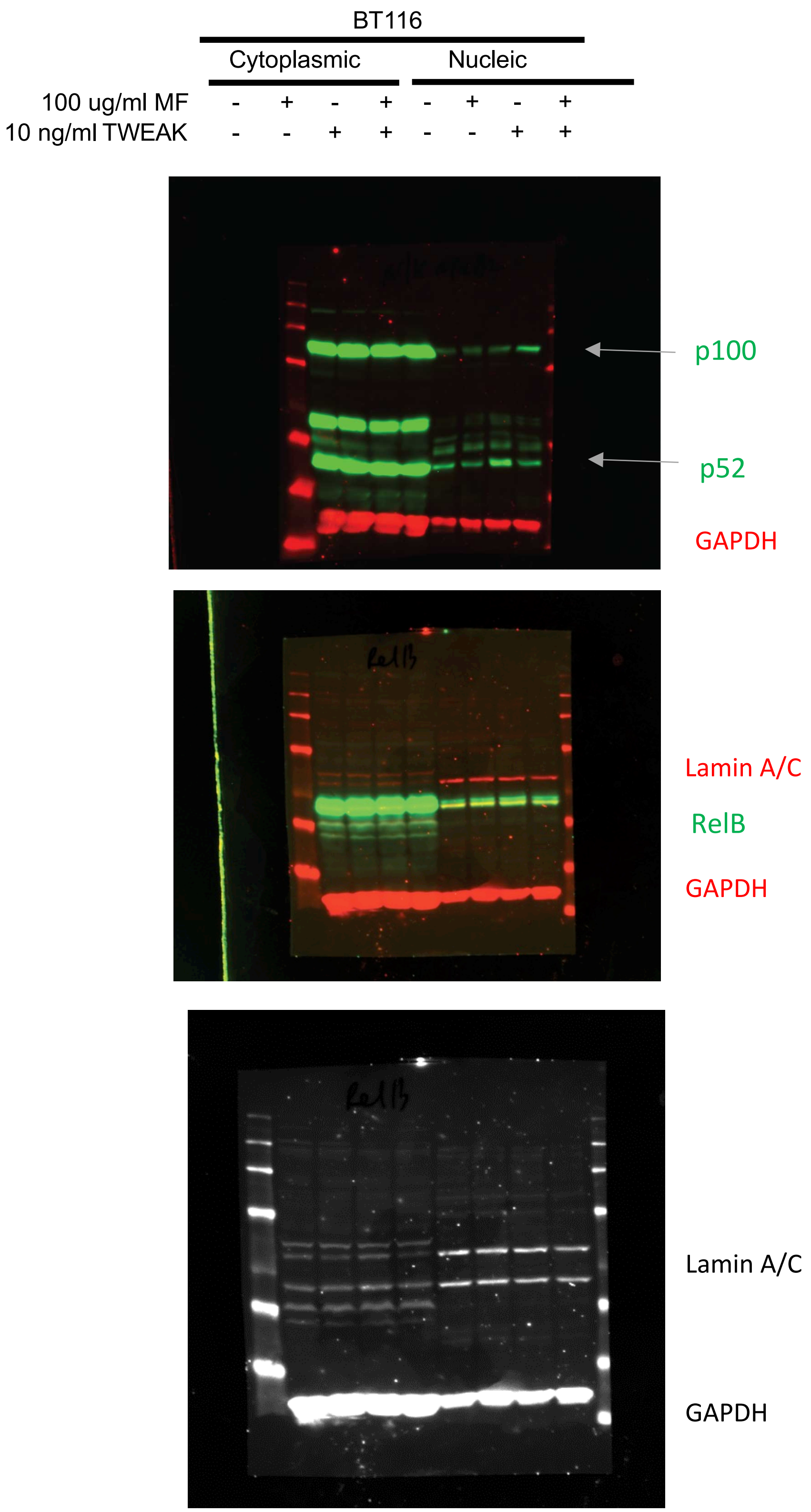

Figure 4A

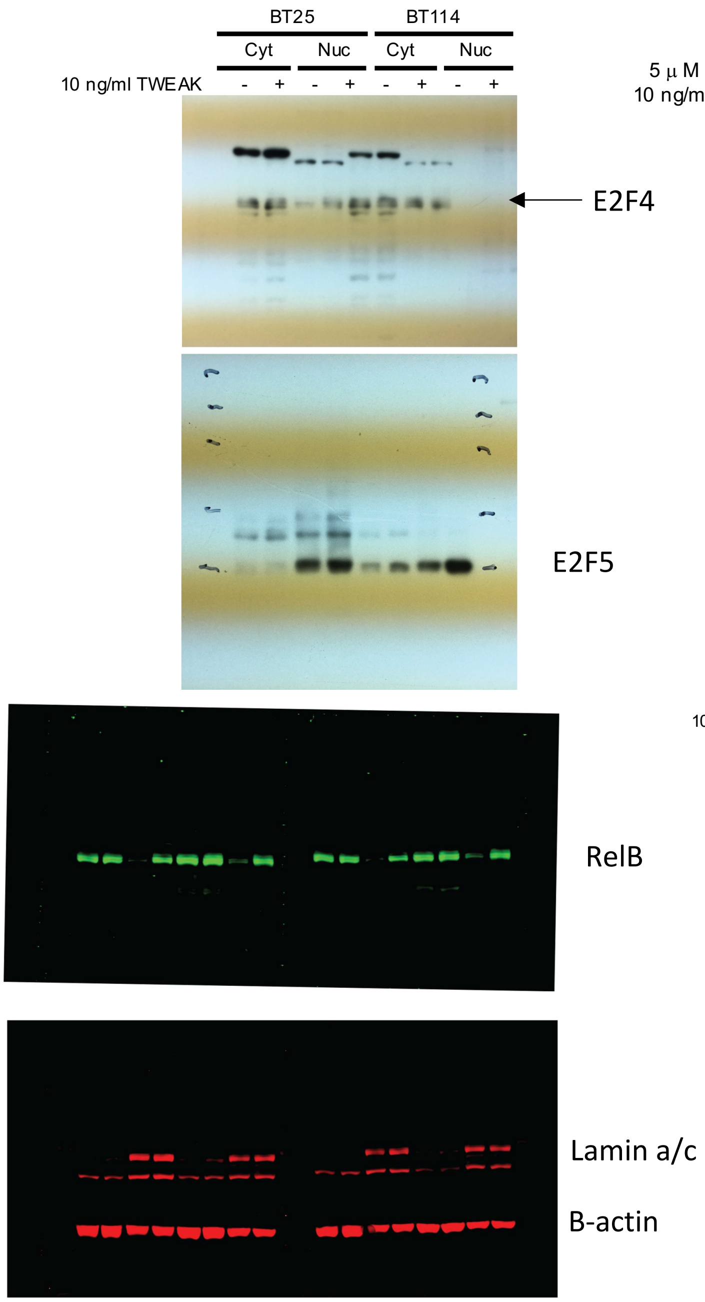


Figure 4B

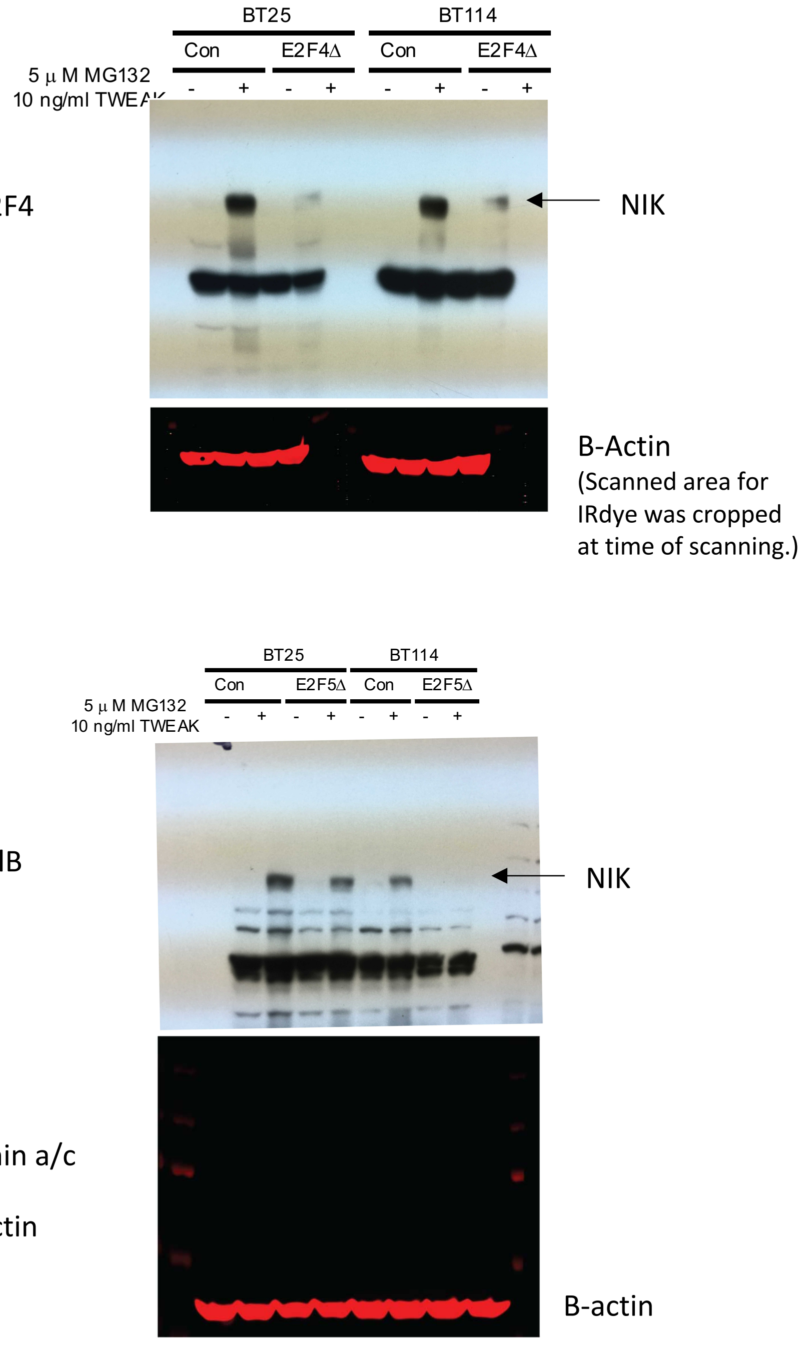


IRdye blot was cropped at time of scanning.

Figure 4B continued

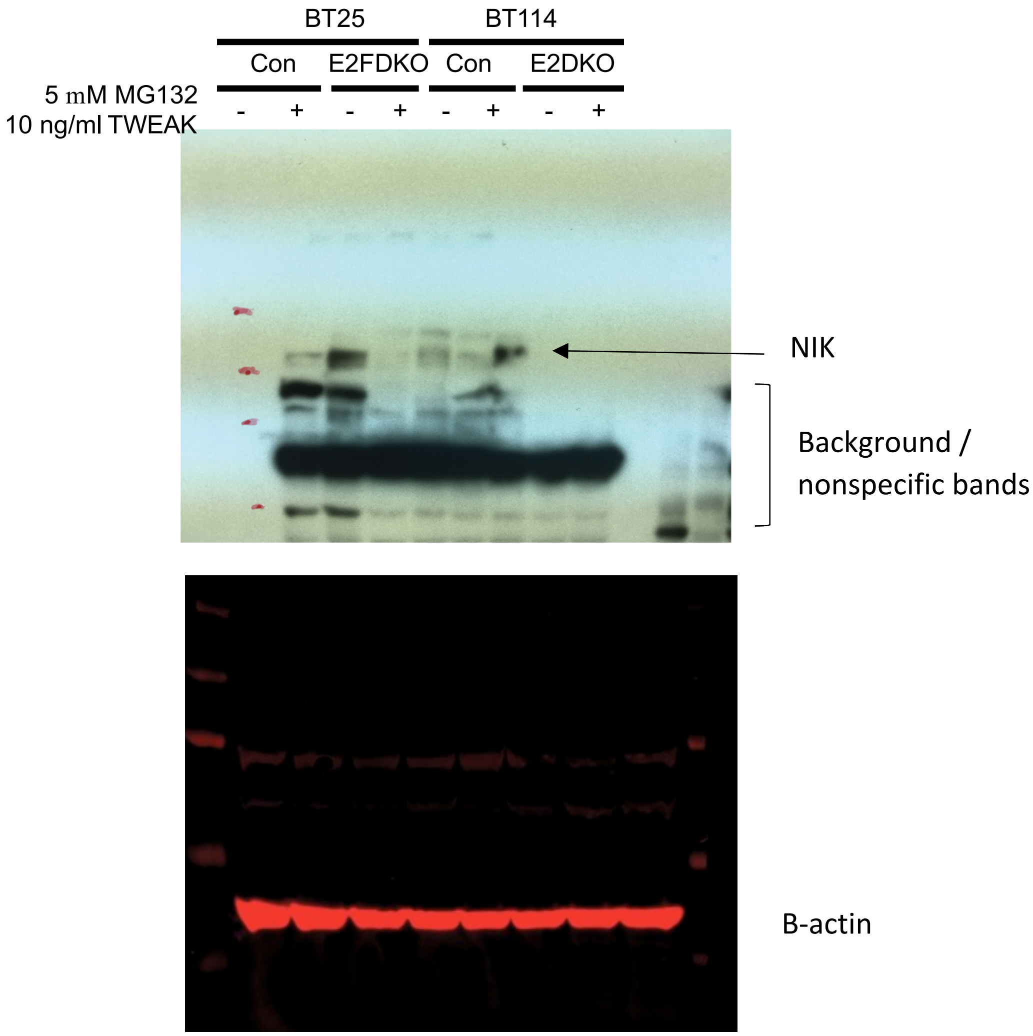

Figure 4C

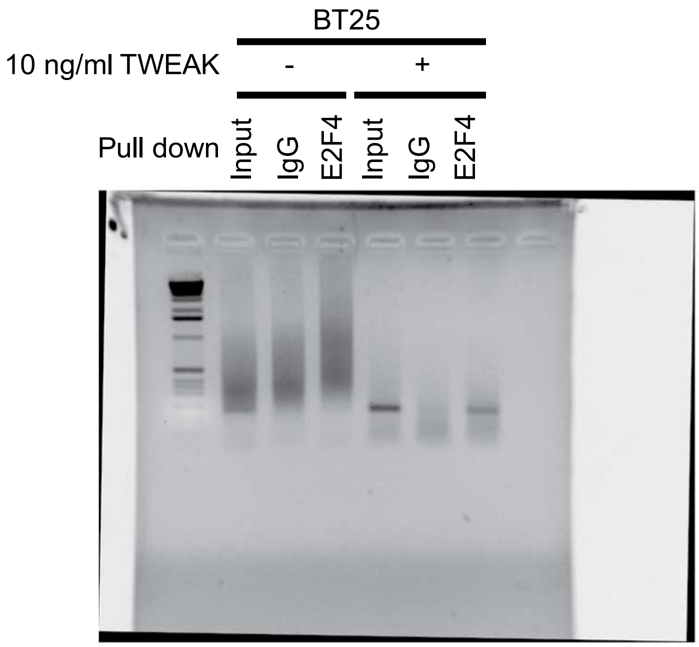
BT25
10 ng/ml TWEAK
-
+
Pull down
Input
IgG
E2F4
Input
IgG
E2F4

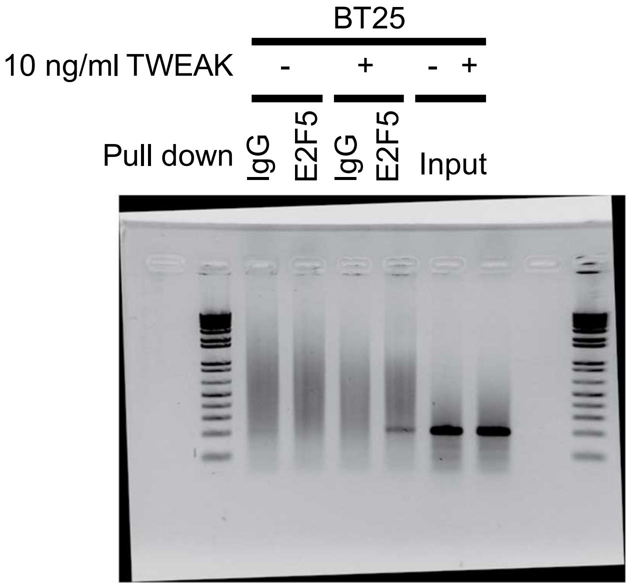
BT25
10 ng/ml TWEAK
-
+
-
+
Pull down
IgG
E2F5
IgG
E2F5
Input

Supplemental Figure 1a

Membrane was cut before antibody probing. Protein ladder marks the boarder of membrane.

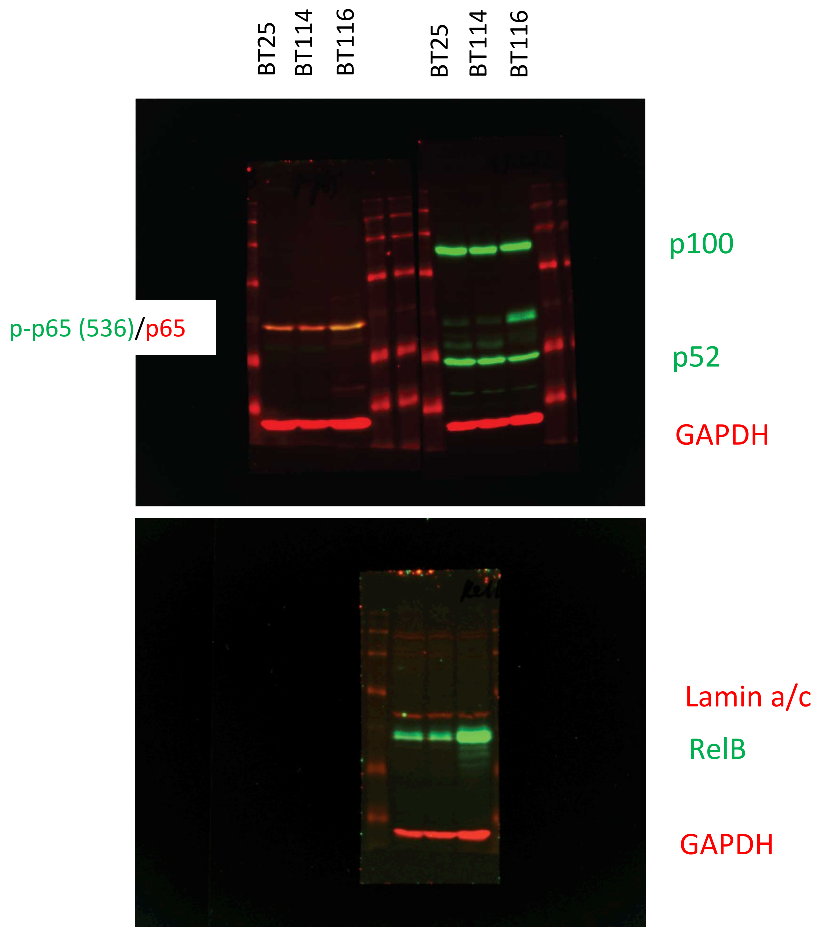

Supplemental Figure 3B

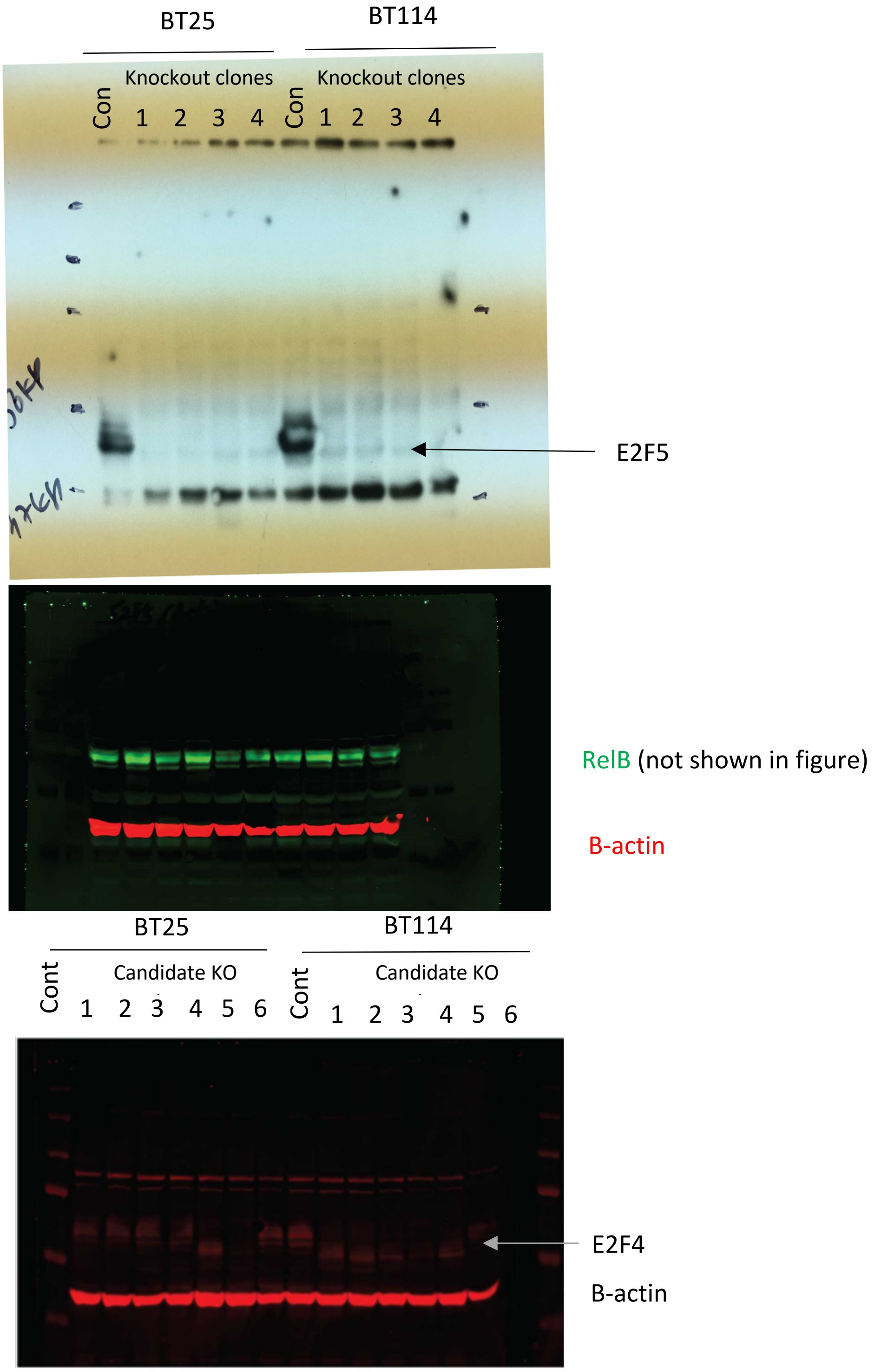

Supplemental Figure 3B continued

Membrane was cut before antibody probing.

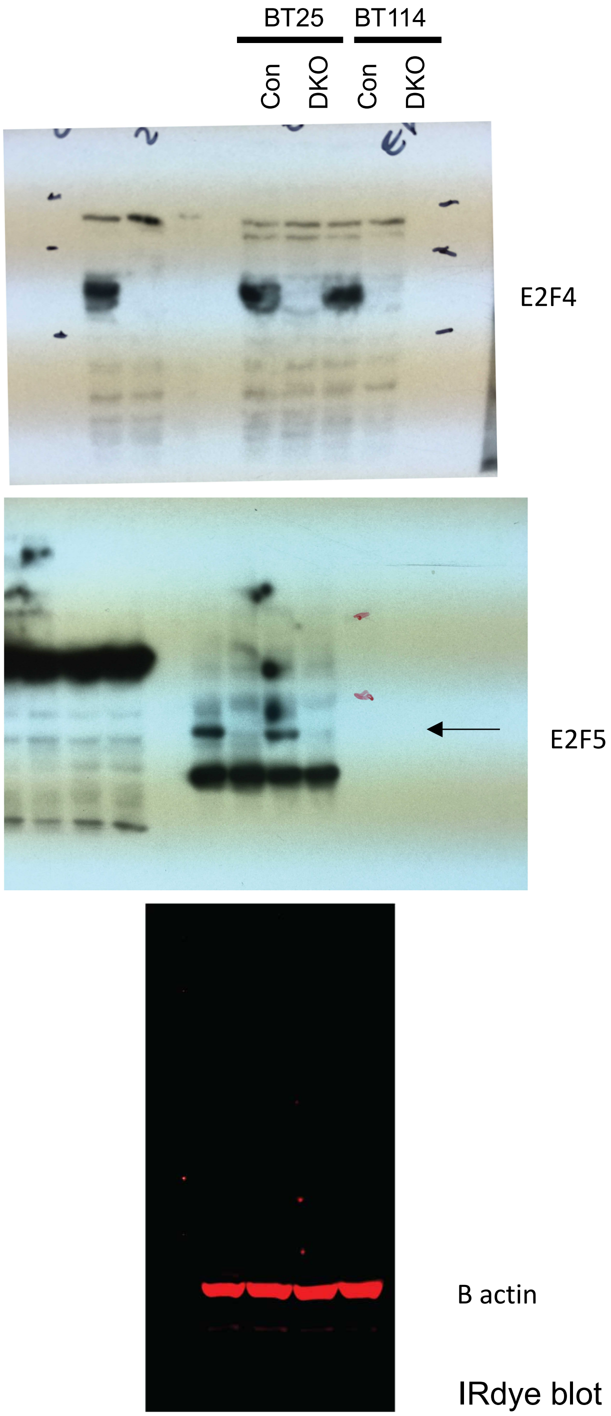


IRdye blot area was selected/cropped at time of scanning. The other lanes were not imaged.

Supplemental Figure 3C

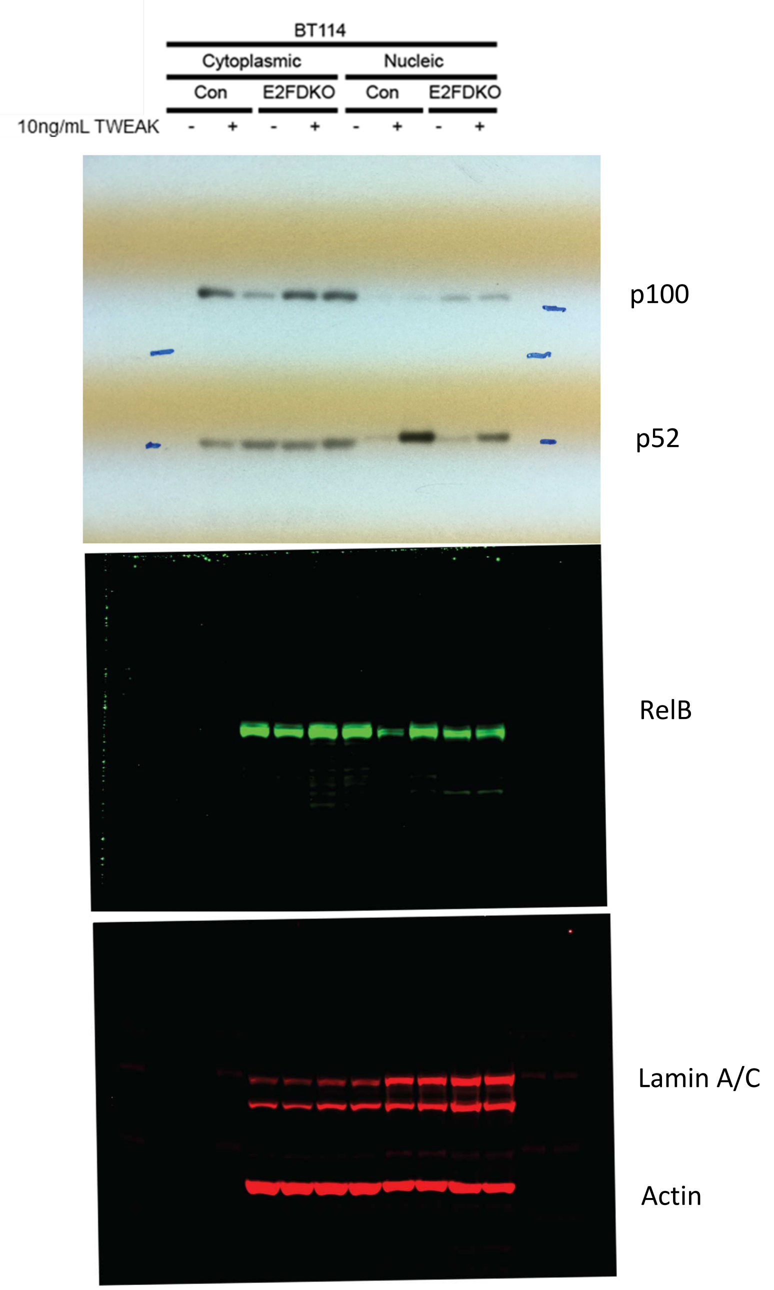

Supplemental Figure 3E

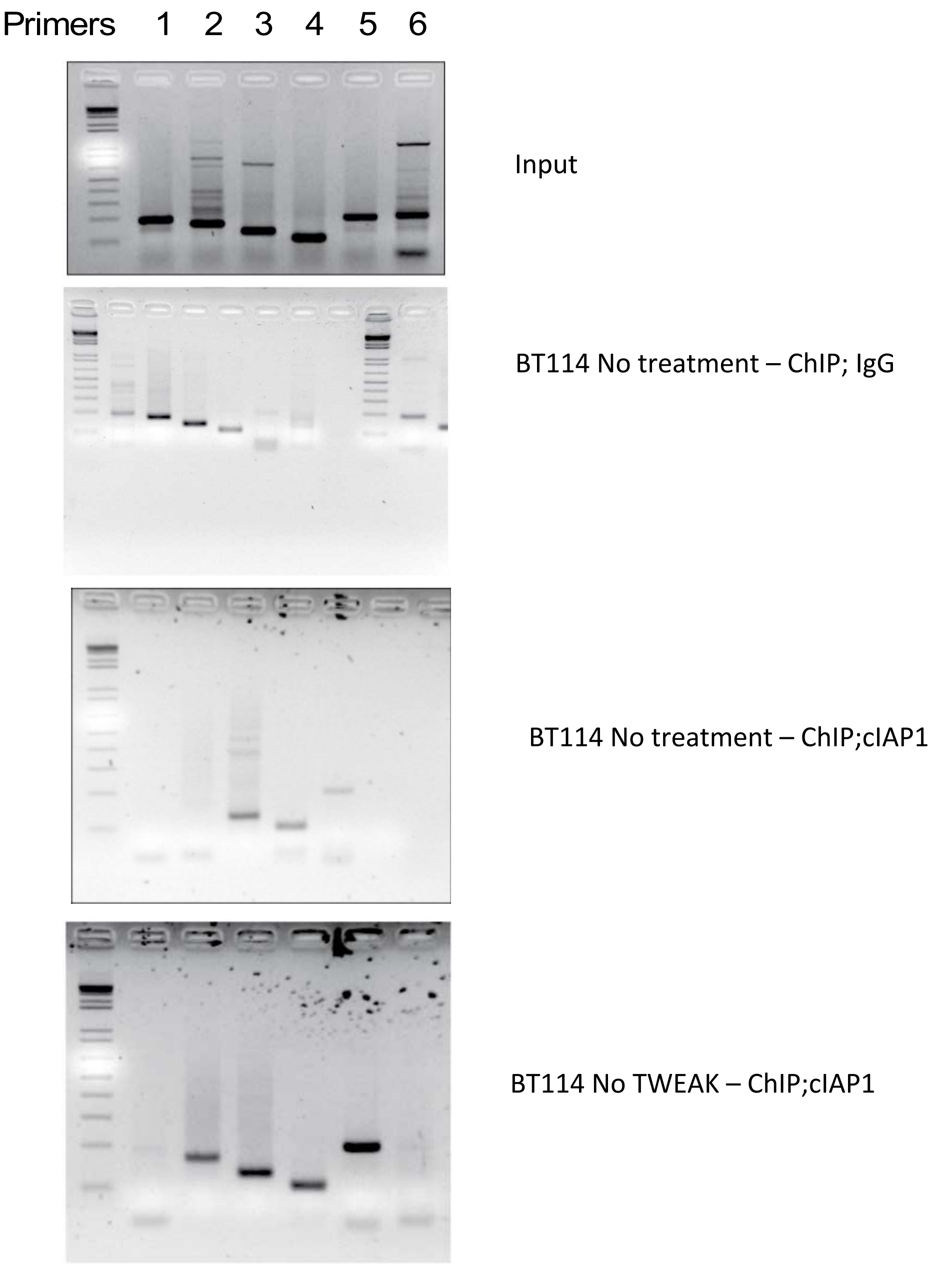

Supplement: Supplementary file 1 — Supplementary Information. [file 41598_2023_38996_MOESM1_ESM.pdf]
